# Detection of Quorum Sensing Activity in the Multidrug-Resistant Clinical Isolate *Pseudomonas aeruginosa* Strain GB11

**DOI:** 10.3390/s140712511

**Published:** 2014-07-11

**Authors:** Huey Jia Cheng, Robson Ee, Yuet Meng Cheong, Wen-Si Tan, Wai-Fong Yin, Kok-Gan Chan

**Affiliations:** 1 Division of Genetics and Molecular Biology, Institute of Biological Sciences, Faculty of Science, University of Malaya, Kuala Lumpur 50603, Malaysia; E-Mails: chenghj90@gmail.com (H.J.C.); robsonee@live.com (R.E.); tmarilyn36@gmail.com (W.-S.T.); yinwaifong@yahoo.com (W.-F.Y.); 2 Jeffrey Cheah School of Medicine & Health Sciences, Monash University Malaysia, Jalan Lagoon Selatan, 46150 Bandar Sunway, Selangor Darul Ehsan, Malaysia; E-Mail: cheong.yuet.meng@monash.edu

**Keywords:** MALDI-TOF, mass spectrometry, *N*-butyryl homoserine lactone (C4-HSL), *N*-hexanoyl homoserine lactone (C6-HSL), *N*-octanoyl homoserine lactone (C8-HSL), *N*-dodecanoyl homoserine lactone (C12-HSL), *N*-3-oxo-dodecanoylhomoserine lactone (3-oxo-C12-HSL), clinical pathogen, cell-to-cell communication

## Abstract

A multidrug-resistant clinical bacteria strain GB11 was isolated from a wound swab on the leg of a patient. Identity of stain GB11 as *Pseudomonas aeruginosa* was validated by using matrix-assisted laser desorption/ionization-time-of-flight mass spectrometry (MALDI-TOF MS). Detection of the production of signaling molecules, *N*-acylhomoserine lactones (AHLs), was conducted using three different bacterial biosensors. A total of four different AHLs were found to be produced by strain GB11, namely *N*-butyryl homoserine lactone (C4-HSL), *N*-hexanoylhomoserine lactone (C6-HSL), *N*-octanoyl homoserine lactone (C8-HSL) and *N*-3-oxo-dodecanoylhomoserine lactone (3-oxo-C12-HSL) using high resolution liquid chromatography tandem mass spectrometry (LC-MS/MS). Of these detected AHLs, 3-oxo-C12-HSL was found to be the most abundant AHL produced by *P. aeruginosa* GB11.

## 1. Introduction

Bacterial cell-to-cell communication (quorum sensing, hereafter QS) modulates the regulation of the network of physiological activities in relation to the population density by synchronizing the concentration of signaling molecules (also known as “autoinducers”) [1,2]. QS is employed by bacteria to sense and response to any changes in the environment through secretion of signaling molecules. When a critical threshold of signaling molecules concentration is achieved, various targeted gene will be regulated either by activating or inhibiting a cascade of gene expression [3]. There are different signaling molecules employed by Gram-negative and Gram-positive bacteria which are *N*-acylhomoserine lactones (AHLs) and oligopeptide molecules, respectively. The QS mechanism utilized by Gram-negative bacteria typically involves a LuxI family protein as “autoinducer” synthase and LuxR family protein as transcriptional activator [4]. QS had been reported in many different types of bacteria to regulate bacterial physiological activities including those phenotypic adaptations to the environment [5–8].

Most Gram-negative pathogenic bacteria possess the ability of producing AHLs that allow them to control virulence genes that could affect bacterial-host interactions [8]. The ability of eukaryotic hosts to produce signals that mimic AHLs suggests that the hosts have adapted and are able to detect the extracellular AHLs [8]. This suggests that the AHL could be the “interkingdom signals” that could make direct interactions between host and bacteria. While *Pseudomonas aeruginosa* is a clinically significant and opportunistic pathogen which often associated with nosocomial infections in compromised hosts, it could be an attractive target for sorting out its communication system and developing QS inhibitors that could act as potential therapeutics [9–12]. *P. aeruginosa* acts as an important pathogen that can express its pathogenicity in almost any part of the human body and can be divided into two general categories which are loss of cell integrity and immune modulation [8]. The creation of biofilms, a bacterial activity regulated by QS with the production of signaling molecules, often renders *Pseudomonas* infections difficult to eradicate and more resistant towards antibiotics [13]. It is known that *P. aeruginosa* adopts a hierarchical network of QS systems to regulate biofilm formation and expression of virulence factors that involves the RhlI/R, LasI/R and PQS signalling systems [14–16]. Thus, investigating the QS molecules in *P. aeruginosa* is an important first step to further understanding this multidrug resistant pathogen and could be one of the means for generating cures for diseases caused by this opportunistic pathogen.

## 2. Experimental Section

### 2.1. Clinical Isolate

The bacterial strain GB11 was isolated from a wound swab taken from an infected leg ulcer of an 83 years old man. Gram stain initially showed scanty leucocytes with numerous gram negative rods and scanty gram positive cocci. Heavy growth of *P. aeruginosa* (initially identified using API^®^ 20 NE) was obtained from the Blood Agar and McConkey Agar plates after overnight incubation. Antimicrobial Susceptibility Testing following the Clinical and Laboratory Standards Institute (CSLI) method was performed [17]. The isolate was sensitive to amikacin, gentamicin, netilmicin, tobramycin and imipenem, but resistant to ceftazidime, cefoperazone and ciprofloxacin.

### 2.2. Bacterial Strains and Culture Conditions

Bacteria strain GB11 was cultured aerobically at 37 °C in Tryptic soy medium (TSm) which consists of casein peptone 15.0 (mg/mL), soy peptone 5.0 (mg/mL), sodium chloride 5.0 (mg/mL), and agar 15.0 (mg/mL). All other strains, including three biosensors and two controls, were cultured aerobically in LB agar and LB medium (LBm) at 28 °C as described previously [18–20], unless stated otherwise. The three biosensors are *Chromobacterium violaceum* CV026 [21], *Escherichia coli* [pSB401] and *E. coli* [pSB1075] [22], which were used for the detection of AHLs while *Erwinia carotovora* GS101 and *E. carotovora* PNP22 act as positive and negative controls, respectively.

### 2.3. Preparation and Screening of AHL by C. violaceum CV026 Biosensor

All AHLs used were obtained as described previously [23–25]. Briefly, AHL stock solutions were prepared with acetonitrile (ACN) (Merck, Frankfurt, Germany) to a concentration of 1 g/L. AHL solutions were stored at −20 °C for not more than a month. The stock solutions were then diluted with ACN to 100 ppm as standard in liquid chromatography mass spectrometry analysis. Production of AHL obtained from strain GB11 was screened using cross-streaking with the biosensor *C. violaceum* CV026 on LBm agar.

### 2.4. AHL Extraction

Bacteria strain GB11 was cultured overnight for 18 h in LBm as illustrated previously [23]. Briefly, 50 mM of 3-[*N*-morpholino] propanesulfonic acid (MOPS) was used to buffer the culture media to pH 5.5 at 37 °C. Extraction of supernatant was conducted twice with equal volume of ethyl acetate buffered with 0.1% v/v glacial acetic acid. AHL extracts were dried completely before further analysis.

### 2.5. Bioluminescence Assay

Bioluminescence assay was conducted with Tecan luminometer (Infinite M200, Männerdorf, Switzerland) as described previously [26,27]. Briefly, 250 μL of diluted *E. coli* biosensor (OD_600_ = 0.1) was used to resuspend the AHL extract. Bioluminescence and optical density (OD_495_) readings were documented simultaneously every 60 min interval for 24 h and experiments were repeated three times with triplicates for each repeat. Bioluminescence measurement was calculated as relative light unit per OD_495_ (RLU/OD_495_) against 24 h [22].

### 2.6. MALDI-TOF MS for Strain Identification

Fresh culture of bacteria strain GB11 was identified by MALDI-TOF (Bruker Daltonik GmbH, Leipzig, Germany) using Bruker FlexControl software version 3.3 (Build 108) as described previously [28].

### 2.7. High Resolution Liquid Chromatography Tandem Mass Spectrometry (LC-MS/MS) Analysis

High Resolution Liquid Chromatography Tandem Mass Spectrometry (LC-MS/MS) was conducted as reported previously [20,24,25]. Briefly, an Agilent 1290 Infinity LC system (Agilent Technologies Inc., Santa Clara, CA, USA) was equipped with a C18 column. Then, 2 μL of sample was injected into the system for analysis. Water and ACN were used as the solvents for mobile phases. HPLC gradient profiles were set as: 0 min: 4:1, 7 min: 1:1, 12 min: 1:4, and 14 min: 4:1. For AHL detection, precursor ion scan mode was used and the product ion *m/z* ratio was set as 102 indicating the [M+H]^+^ ion of the core lactone ring where the precursor ions were then scanned from 150 to 400. Thus, various AHLs were identified based on the detection of the fragmentation of the core HSL moiety in the collision cell.

## 3. Results and Discussion

### 3.1. Detection of AHL Production in Strain GB11

The ability of a variety of bacterial biosensors has paved a pathway for researching and understanding bacterial QS properties and be able to provide a fast and accurate way for detecting the production of AHLs. The AHL biosensor practically relies on the LuxR protein and displays a specific attraction towards the cognate AHL and positively regulates the transcriptional of targeted gene [29]. CV026 biosensor is a white colony *C. violaceum* mutant [21]. It is most sensitive to C6-HSL, its natural AHL produced by wild-type *C. violaceum*, however it can also detect short-chain AHLs ranging from 4 to 8 carbon side-chain AHLs with or without C-3 substitution. Although the CV026 bioassay is comparatively easy to perform, false-negative results occasionally happen when some sample isolates produce bactericides that might kill CV026 [22]. Hence, bioassays using CV026 should be coupled with other analytical methods such as mass spectrometry. Also, other types of AHL biosensors should be used in addition to CV026.

**Figure 1.**
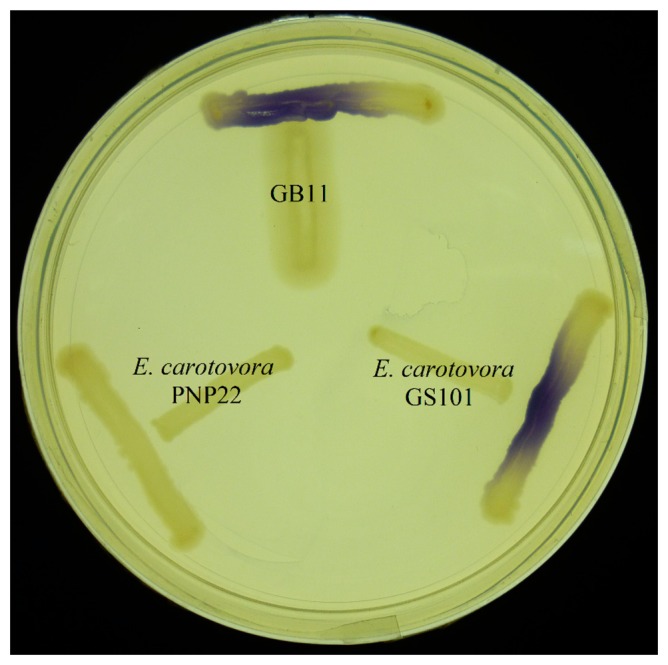
Detection of AHL by *C. violaceum* CV026. Purple pigmentation was visible after 24 h of incubation as the AHLs produced by the isolate GB11 diffused through the agar and activated the biosensor strain. *E. carotovora* GS101 and *E. carotovora* PNP22 were included as positive and negative controls, respectively.

**Figure 2.**
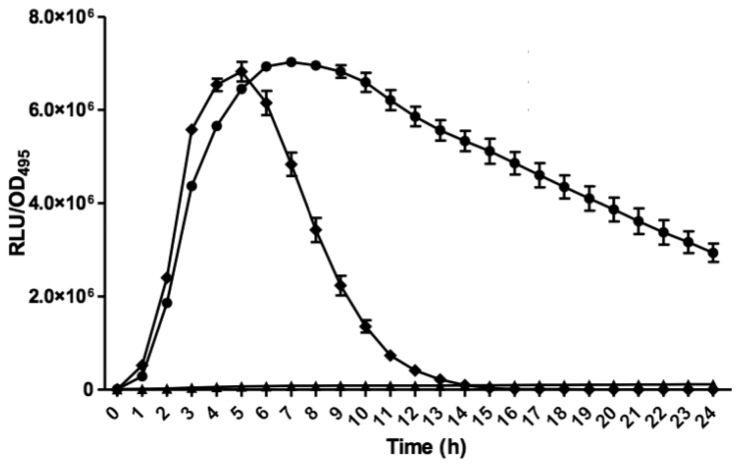
Measurement of bioluminescence with *E. coli* [pSB401] and E.coli [pSB1075] AHL biosensors. Increase in bioluminescence measurement in both *E. coli* [pSB401] (circle) and *E. coli* [pSB1075] (diamonds) indicates the production of both short and long-chain AHLs in the spent supernatant of strain GB11, respectively. Extract from uninoculated LB broth was used as negative control (triangles). Each point represents a mean value of three independent replicates.

*E. coli* harbouring the pSB401 and pSB1075 plasmids are chosen as alternative AHL biosensors. Plasmid pSB401 is a pACYC184 plasmid containing a fusion of *luxRluxl*'∷*luxCDABE* while plasmid pSB1075 is a pUC18 plasmid containing *lasRlasl*'∷*luxCDABE* whereby *E. coli* [pSB401] and *E. coli* [pSB1075] are capable of detecting short-chain AHLs and long-chain AHLs, respectively [22]. Strain GB11 showed induction of purple violacein in CV026 biosensor after incubation for 24 h that indicated the presence of short-chain AHLs (Figure 1). Bioluminescene bioassays were conducted to validate this results with increase of bioluminescence activity in both *E. coli* [pSB401] and *E. coli* [pSB1075] (Figure 2).

### 3.2. Identity of Strain GB11

**Figure 3.**
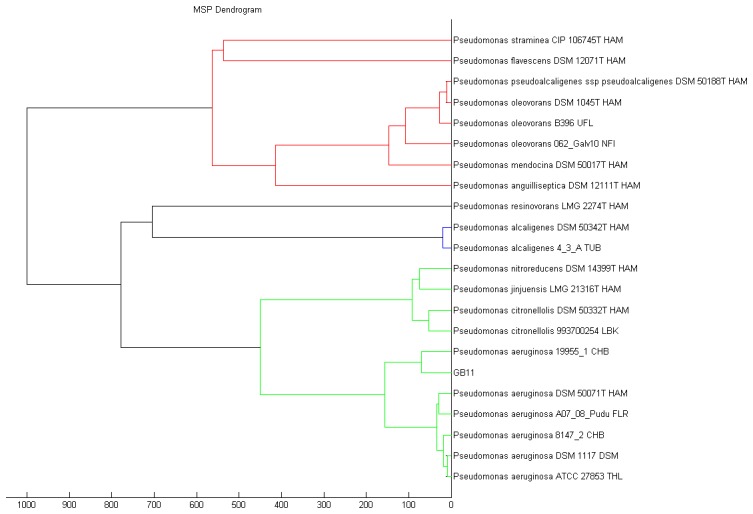
Bacterial identification of strain GB11 using MALDI-TOF. Dendogram shows the distance value assembled from the reference between species in the database using logarithmic function.

**Figure 4.**
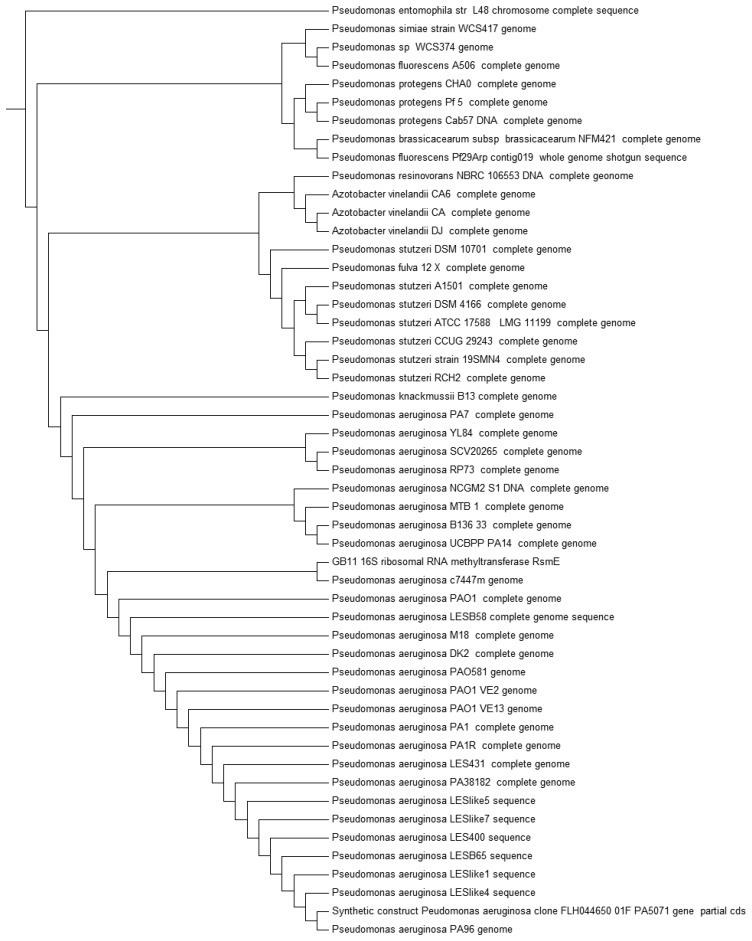
Bacterial identification of strain GB11 using 16S rRNA gene phylogenetics analysis. Phylogeny tree shows the 16S rRNA gene of strain GB11 clustered closely with *P. aeruginosa* strains. All sequences data are obtained from NCBI database.

Recent advances ease the path on bacteria identification with established technique such as MALDI-TOF, which is one of the fastest mean to classify bacteria [30,31]. Strain GB11 was identified as *Pseudomonas aeruginosa* as the best match in MALDI-TOF identification (Figure 3). Our MALDI-TOF results is in agreement with the preliminary microbiological assessment of strain GB11. Later, we have verified the identity of strain GB11 using 16S rRNA gene analysis where a phylogeny tree was constructed by comparing the closely-related sequences from NCBI database (Figure 4).

### 3.3. AHL Profile Analysis by High Resolution Liquid Chromatography Tandem Mass Spectrometry (LC-MS/MS)

*P. aeruginosa* is an opportunistic pathogen that often infects patients with immunocompromised conditions such as those with cancers, burns or cystic-fibrosis [32]. QS is believed to be adopted by *P. aeruginosa* to invade and infect its host. The expression of many genes in *P. aeruginosa* are known to be regulated by QS such as *lasA* (onset of proteolysis and elastolysis) [33] and *toxA* [34]. Thus, inhibition of *P. aeruginosa* QS had been proposed as the promising alternative to attenuate its virulence and biofilm formation [35–37]. Understanding the types of AHLs produced by *P. aeruginosa*, is a milestone bringing us closer to finding effective ways to treat *P. aeruginosa* infections.

**Figure 5.**
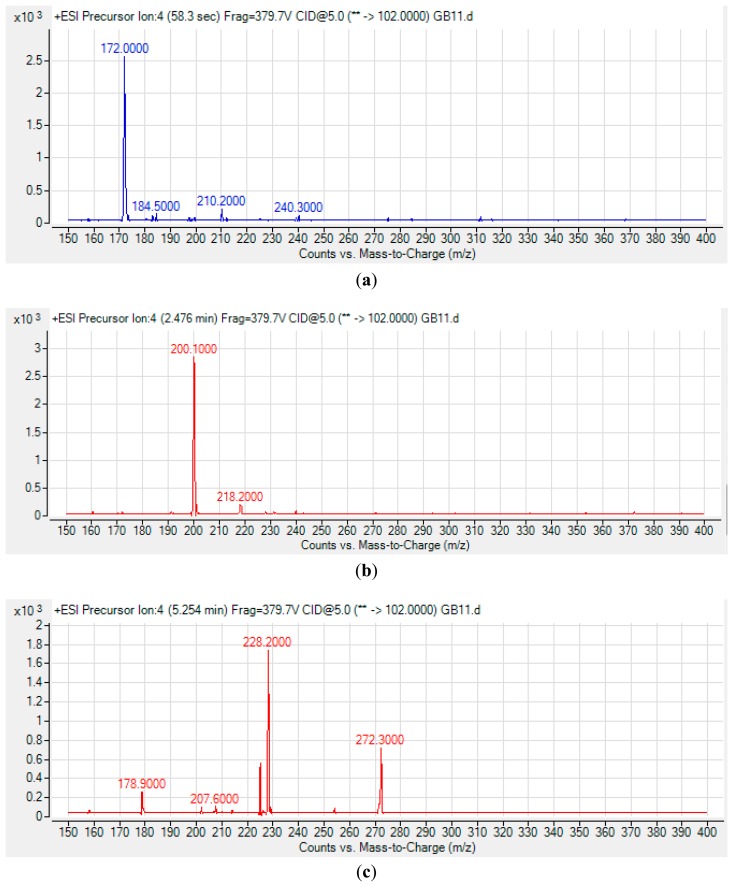

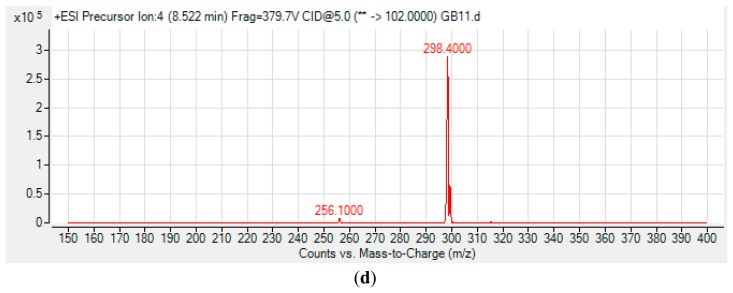
Mass spectrometry analysis of AHLs produced by *Pseudomonas aeruginosa* GB11. (**a**) C4-HSL (Retention time: 58.3 s; *m/z*: 172.0000 Abundance: 2565.42); (**b**) C6-HSL (Retention time: 2.4764 min; *m/z*: 200.1000; Abundance: 2855.4); (**c**) C8-HSL (Retention time: 5.254 min; *m/z*: 228.2000; Abundance: 1742.76); (**d**) 3-oxo-C12-HSL (Retention time: 8.522 min; *m/z:* 298.4000; Abundance: 288910.28).

The spent culture of GB11 strain was analyzed using LC-MS/MS and this confirmed that *P. aeruginosa* GB11 produced four different types of AHL molecules which are *N*-butyryl-L-homoserine lactone (C4-HSL), *N*-hexanoyl-L-homoserine lactone (C6-HSL), *N*-octanoyl-L-homoserine lactone (C8-HSL) and *N*-(3-oxododecanoyl)-L-homoserine lactone (3-oxo-C12-HSL) (Figure 5). Of these detected AHLs, 3-oxo-C12-HSL is the most abundant AHL produced by *P. aeruginosa* GB11 and C8-HSL being the least produced AHL by this isolate. To our best knowledge, this is the first report on multiple production of these AHLs by *P. aeruginosa*. We believed that the investigation on AHL profile of *P. aeruginosa* GB11 would be important to understand the QS mechanism particularly virulent determinants regulation in this multidrug resistant clinical isolate.

## 4. Conclusions

QS plays a vital role in bacteria by regulating their physiological activities so that they are able to adapt to the environment and threaten their hosts. This work illustrated the importance in expanding the research on AHL-producing pathogens isolated from human wounds.
